# Cloning, Expression, Purification, and Characterization of Glutaredoxin from Antarctic Sea-Ice Bacterium *Pseudoalteromonas* sp. AN178

**DOI:** 10.1155/2014/246871

**Published:** 2014-07-07

**Authors:** Quanfu Wang, Yanhua Hou, Yonglei Shi, Xiao Han, Qian Chen, Zhiguo Hu, Yuanping Liu, YuJin Li

**Affiliations:** ^1^School of Marine and Technology, Harbin Institute of Technology, Weihai 264209, China; ^2^Shandong Provincial Engineering Technology Research Center of Marine Health Food (Taixiang Group), Rongcheng 264300, China; ^3^Shandong Provincial Key Laboratory of Processing Technology of Frozen Prepared Food (Taixiang Group), Rongcheng 264300, China

## Abstract

Glutaredoxins (Grxs) are small ubiquitous redox enzymes that catalyze glutathione-dependent reactions to reduce protein disulfide. In this study, a full-length Grx gene (*Ps*Grx) with 270 nucleotides was isolated from Antarctic sea-ice bacterium *Pseudoalteromonas* sp. AN178. It encoded deduced 89 amino acid residues with the molecular weight 9.8 kDa. Sequence analysis of the amino acid sequence revealed the catalytic motif CPYC. Recombinant *Ps*Grx (r*Ps*Grx) stably expressed in *E. coli* BL21 was purified to apparent homogeneity by Ni-affinity chromatography. r*Ps*Grx exhibited optimal activity at 30°C and pH 8.0 and showed 25.5% of the activity at 0°C. It retained 65.0% of activity after incubation at 40°C for 20 min and still exhibited 37.0% activity in 1.0 M NaCl. These results indicated that r*Ps*Grx was a typical cold active protein with low thermostability.

## 1. Introduction

Glutaredoxins (Grxs) are ubiquitous small disulfide oxidoreductases and members of the thioredoxin (Trx) fold superfamily. They catalyze the reduction of protein disulfides and of glutathione- (GSH-) protein mixed disulfides in a coupled system with GSH, NADPH, and glutathione reductase (GR) [1]. Grxs were first discovered in* E. coli* as a hydrogen donor for ribonucleotide reductase and regulated protein activity by reversibly reducing disulfide bonds in their targets to achieve their role in antioxidative response [2]. Subsequently, Grxs have been isolated and identified from different organisms, including* E. coli*,* Saccharomyces cerevisiae*,* Chlamydomonas reinhardtii*,* Synechocystis *PCC 6803,* Oryza sativa*,* Populus trichocarpa*, and human Homo sapiens [3–6]. For example, in yeast 8 members of Grxs have been found, named Grx1 to Grx8 in chronological order of identification [3, 5]. 31 members of Grxs in* Arabidopsis*, 4 members of Grxs in* E. coli*, and 3 Grxs members in humans were discovered and studied [7]. Grxs could participate in a variety of cellular functions, such as providing reduction of equivalents for ribonucleotide reductase, antioxidant defense, control of cellular redox state, and the redox control of transcription and signal transduction [8, 9]. Thus Grxs from different species, or even different strains of the same species, may differ in their structural, catalytic, and functional properties.

Antarctic sea-ice is considered the unique, mostly pristine, and extreme environment. Microbes living within the sea-ice have a high degree of biochemical and physiological adaptation to low temperature, high level of dissolved oxygen, and changeable salinity conditions [10]. Several studies have shown that such extreme environments can induce excessive accumulation of reactive oxygen species (ROS), which will damage macromolecules and thus change normal signal conduction in Antarctic microbes [11, 12]. To cope with such oxidative damage and insure normal signaling events, Antarctic microbes have developed complex and precisely controlled antioxidant systems by regulating cellular gene expression which enables organisms to maintain proteins and other cellular components as usual. Antarctic fungi* Penicillium* sp. could increase activities of antioxidant enzymes such as catalase and superoxide dismutase for adaptation to the high oxygen concentration [13]. Thioredoxin and thioredoxin reductase from the Antarctic psychrophilic eubacterium* Pseudoalteromonas haloplanktis* were investigated through the heterologous expression of their genes and the biochemical investigation on the recombinant proteins [14]. Our recent studies have suggested that glutathione S-transferase (GST) can play a major role in a coordinated protection mechanism against low temperature in Antarctic sea-ice bacterium* Pseudoalteromonas* sp. [15]. Glutaredoxins, as antioxidant proteins, were known to be involved in ROS elimination and cellular oxidative-reductive balance [16]. Thus, Antarctic sea-ice microorganisms would be the new and potential sources of oxidative stress-inducible enzymes. To our knowledge, Grxs from Antarctic bacteria have been not biochemically characterized. The present work reports the molecular cloning, expression, and characterization of a novel Grx from sea-ice bacterium* Pseudoalteromonas* sp. AN178.

## 2. Materials and Methods

### 2.1. Bacteria Cultivation and Collection

Strain AN178 was identified as* Pseudoalteromonas* sp. based on 16S rRNA gene sequence. It was isolated from Antarctic sea-ice (68°30′E, 65°00′S) and was used as a source of the gene encoding Grx. Vector pET-28a (+) and* E. coli* BL21(DE3) were used for Grx gene cloning and expression.

Strain* Pseudoalteromonas* sp. AN178 was inoculated in the 2216E medium (peptone 0.5%, yeast extract 0.1%, pH 7.5, made by natural sea water) with shaking at 120 rpm and 8°C.* E. coli* strains containing recombinant plasmids were cultured in Luria-Bertani (LB) medium containing kanamycin (100 mg/L).

### 2.2. DNA Manipulation and Cloning of* Ps*Grx

The genomic DNA was isolated by using a Genomic DNA Prep Kit (Tiangen Biotech Co., Ltd., Beijing, China) according to the manufacturer's instructions. DNA purification was done using Gel DNA Purification Kit Ver.2.0 (Tiangen Biotech Co., Ltd., Beijing, China). One primer set (5′-AGGAATATRATGAGTAATGTTGTBTTAT-3′, 5′-ATTATAYTTAGTTAAATTTAAGCGTTBAGT-3′) was designed based on the nucleotide sequences immediately upstream and downstream the known coding sequences of Grx from the genera* Pseudoalteromonas*. PCR reaction was carried out with the following protocol: denaturation at 94°C for 5 min, followed by 30 cycles of denaturation at 95°C for 1 min, annealing at 47°C for 1 min, extension at 72°C for 1.5 min, and a 10 min final extension at 72°C.

### 2.3. Sequence Analysis

The open reading frame (ORF) for the* Ps*Grx gene was determined using ORF finder (http://www.ncbi.nlm.nih.gov/gorf/) and translated into the corresponding amino acid sequence. Protein identity and amino acid sequence of* Ps*Grx were determined by using the BLAST tool in the NCBI website and the conserved amino acids were also determined by using ClustalW program (http://www.ebi.ac.uk/Tools/clustalw2/index.html). Theoretical* p*I values and predicted molecular masses were calculated using Prot-Param tools (http://kr.expasy.org/tools/protparam.html).

### 2.4. Expression and Purification of the* Ps*Grx

The open reading frame of the DNA encoding* Ps*Grx was amplified by PCR with the upstream primer 5′-CAGGGATCCATGAGTAATGTTGTTTT-3′ (the* Bam*HI site was underlined) and the downstream primer 5′-TATAAGCTTAGCGTTTAGTGTGGCAT-3′ (the* Hin*dIII site was underlined). The PCR amplified fragment was digested with* Bam*HI and* Hin*dIII and cloned into the expression vector pET-28a (+) to construct pETgrx. After checking the correct sequences, the recombinant vector was introduced into* E. coli* BL21(DE3).

Grx-fused protein was expressed in* E. coli*. Positive clones were screened on LB broth containing kanamycin (100 mg/L) and grown with shaking at 200 rpm for overnight at 37°C. IPTG (final concentration of 0.12 mg/mL) was added to the medium at the A_600_ value of 0.6–0.8. After additional cultivation at 37°C for 5 h, the cells were harvested by centrifugation (7500 ×g for 15 min at 4°C) and washed with an appropriate volume of sterile water.

Recombinant* Ps*Grx (r*Ps*Grx) was mainly expressed as inclusion bodies. After the cells were homogenized by ultrasonic treatment, the inclusion bodies were collected by centrifugation (12,000 ×g for 10 min at 4°C). Inclusion bodies were dissolved by the binding buffer (8 mM Urea, 5 mM imidazole, 500 mM NaCl, and 20 mM Tris-HCl) at 5 mL per gram wet weight. The cell suspension was stirred for 60 min at room temperature, and the soluble fraction was collected by centrifugation (12,000 ×g for 15 min at 4°C). The soluble fraction was loaded to the Ni-NTA resin affinity chromatography, following the manufacturer's recommendations. The protein was eluted with an imidazole gradient (40, 100, and 250 mM) with five column volumes at a flow rate of 1.0 mL/min. Purified protein was dialyzed by PBST containing gradient decreasing concentrations of 6, 4, 2, and 0 M urea. After a thorough dialysis the solution protein was collected and stored at 4°C. 12.5% SDS-PAGE was applied for confirmation of the expressed product. The protein concentration was measured by Coomassie Brilliant Blue G-250 method using bovine serum albumin as the standard.

### 2.5. Grx Activity Assay

Grx activity was performed as described by monitoring the consumption of NADPH at 340 nm during the GSH-dependent reduction of 8 mM *β*-hydroxyethyl disulfide (HED) using 1 mM GSH coupled with of 0.6 units of yeast GR (Sigma) [17]. One unit of Grx activity was defined as the amount required to oxidize 1 *μ*M of NADPH per min at 25°C.

### 2.6. Biochemical Properties of r*Ps*Grx

The optimal temperature for r*Ps*Grx in the range of 0°C to 60°C was determined in Tris-HCl (pH 7.0) over 10 min using the standard assay method. The thermal stability for r*Ps*Grx was measured by incubating the protein at 40°C and 50°C for 10, 20, 30, and 40 min in Tris-HCl (pH 7.0), respectively and then directly put into an ice water bath and the residual activity was measured as described by using the standard assay. The optimal pH for r*Ps*Grx was determined at 25°C in the range of pH 5.0 to 10.0 by using the following buffers: sodium acetate/acetic acid (pH 5.0), NaH_2_PO_4_/Na_2_HPO_4_ (pH 5.0, 6.0, 7.0, and 8.0), and Tris-HCl (pH 8.0, 9.0, and 10.0). Effects of metal ions and various other agents on the Grx activity were also investigated in the standard assay, and the concentration of each reagent was indicated in Table 1. For this, the protein was incubated with various compounds in 50 mM Tris-HCl (pH 7.0) at 20°C for 1 h. Residual activity was determined under optimal protein assay conditions. Activity assayed in the absence of any additives was taken as 100%. The kinetic properties for the r*Ps*Grx were determined at varying concentrations of HED (0.4 to 8 mM) with a fixed concentration of GSH (1 mM). Three independent experiments were performed at each substrate concentration, and the apparent *K*_*m*_ and *V*_max⁡_ were calculated from Lineweaver-Burk plots.

## 3. Results

### 3.1. Gene Cloning and Sequence Analysis

**Figure 1:**
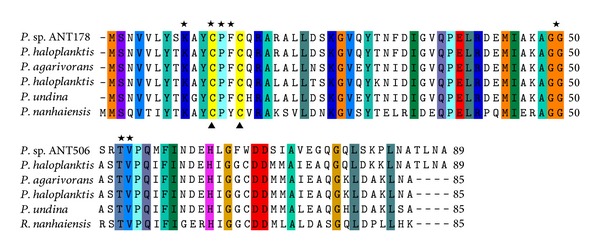
Alignment of amino acid sequences of* Ps*Grx with the sequences of other Grxs. The displayed sequences are* Pseudoalteromonas haloplanktis* Grx (YP_338909),* Pseudoalteromonas agarivorans* Grx (WP_004588615),* Pseudoalteromonas haloplanktis* Grx (WP_016708488),* Pseudoalteromonas undina* Grx (WP_010391834), and* Rheinheimera nanhaiensis* Grx (WP_008217797). The shaded boxes in same color indicate identical residues. Symbols: closed triangles, cysteines (C) in the active site; pentagrams, residues involved in glutathione-binding site.

A full-length Grx gene (Genbank accession no: KF361316) from* P*. sp. AN178, designated* Ps*Grx, was composed of 270 nucleotides encoding 89 amino acid residues. The calculated molecular weight and isoelectric point of* Ps*Grx were 9.8 kDa and pH 5.2, respectively. In the amino acid sequence of* Ps*Grx, the total number of negatively charged residues (Asp + Glu) was 10, while the total number of positively charged residues (Arg + Lys) was 8. Multiple sequence alignment revealed the conserved GSH binding site (G-site) (Lys^9^, Cys^12^, Pro^13^, Phe^14^, Gly^50^, Thr^53^, and Val^54^), and catalytic residues (Cys^12^ and Cys^15^) were identified in the sequence (Figure 1). The blast search in the NCBI GenBank using the deduced amino acid sequence of* Ps*Grx revealed that it had high sequence similarity to Trx-superfamily. The amino acid sequence of* Ps*Grx shared 85.6%, 74.4%, 74.4%, 72.2%, and 50.0% identity with* P. haloplanktis* TAC 125 Grx (YP_338909),* P. agarivorans* Grx (WP_004588615),* P. haloplanktis* Grx (WP_016708488),* P. undina* Grx (WP_010391834), and* R. nanhaiensis* Grx (WP_008217797), respectively. This indicated that* Ps*Grx could be a novel Grx belonging to Trx-like superfamily.

### 3.2. Expression and Purification of the* Ps*Grx

**Figure 2:**
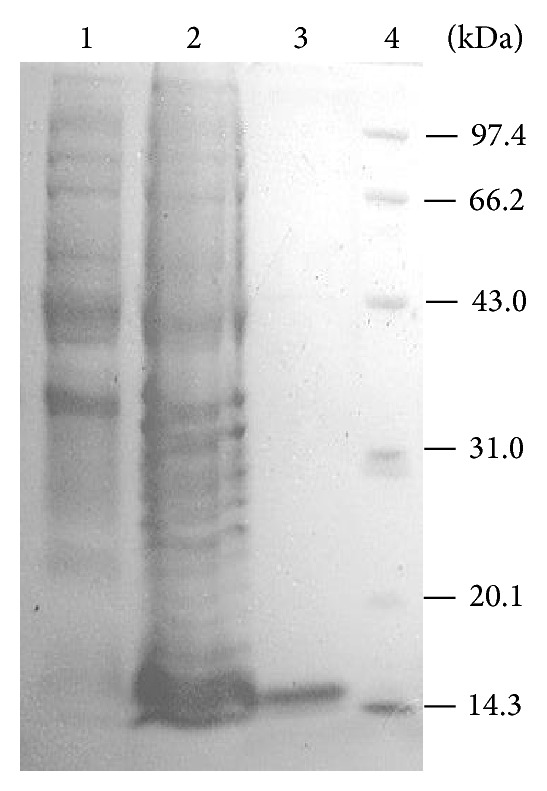
SDS-PAGE of the expression and purification of* Ps*Grx in* E. coli*. lane 1: IPTG-induced* E. coli* BL21 (Grx^−^); lane 2: a total cell lysate of IPTG-induced* E. coli* BL21 (Grx^+^); lane 3: purified* Ps*Grx; lane 4: molecular weight protein marker.

The recombinant vector pETgrx was constructed and transformed into* E. coli* BL21(DE3). r*Ps*Grx was overexpressed by IPTG induction, and SDS-PAGE results showed a strong band approximately 15.0 kDa was found compared to uninduced cells (Figure 2). The purification process resulted in a 5.81-fold purification with 43.83% final recovery and 53.25 U/mg specific activity.

### 3.3. Biochemical Properties of r*Ps*Grx

**Figure 3:**
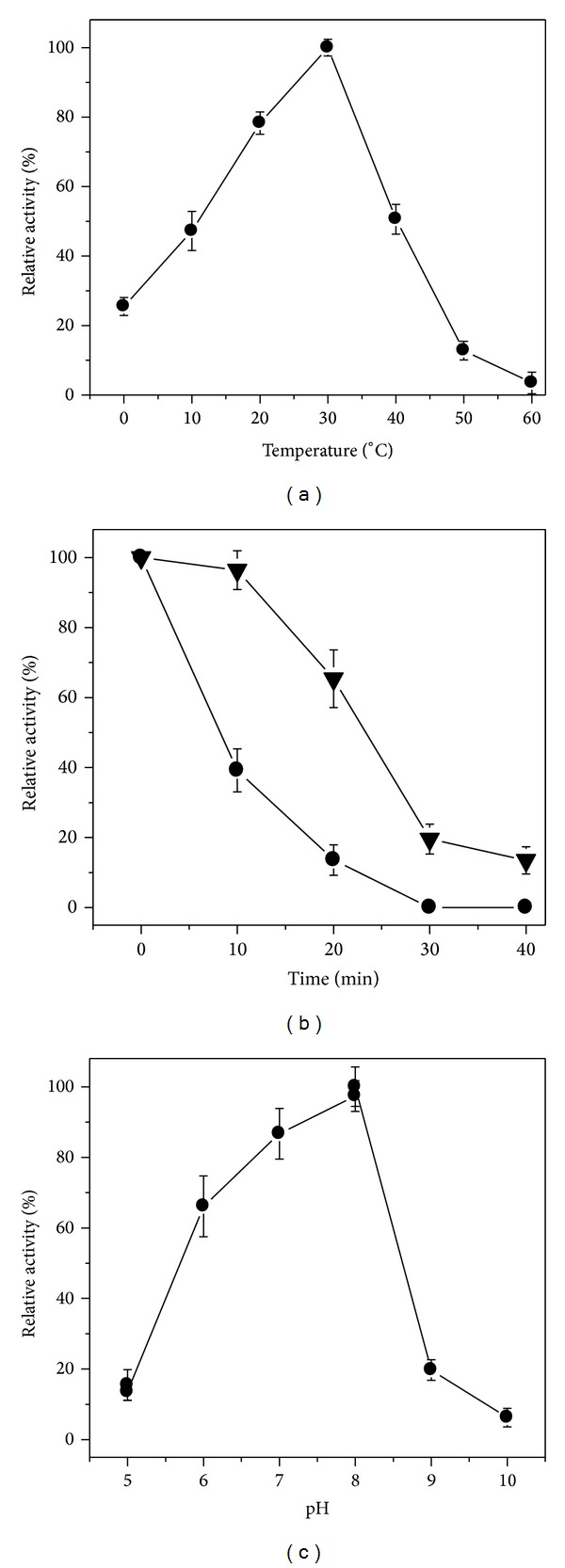
Biochemical properties of the r*Ps*Grx. (a) Effect of temperatures on activity of the r*Ps*Grx. (b) Effect of temperatures on the stability of the r*Ps*Grx. The protein was incubated at 40°C closed triangles and 50°C closed circles for 10, 20, 30, and 40 min, respectively and then directly put into an ice water bath and the residual activity was measured in the standard assay. (c). Effect of pH on activity of the r*Ps*Grx.

Effects of temperatures on the r*Ps*Grx activity were measured in temperature range of 0°C–60°C (Figure 3(a)). The optimal temperature for r*Ps*Grx activity was 30°C. r*Ps*Grx showed about 47.2% and 25.5% of its activity at 10°C and 0°C, respectively. As for the thermal stability assay, r*Ps*Grx was evaluated by incubation at temperature 40°C and 50°C for 40 min (Figure 3(b)). r*Ps*Grx showed 65.0% of its activity after incubation at 40°C for 20 min while lost almost all the activity after incubation at 50°C for 30 min. Effect of pH on the Grx activity was studied using different pH buffers. As shown in Figure 3(c), r*Ps*Grx showed activity in a broad pH range of 5.0–9.0. The maximal activity was observed at pH 8.0. But it was completely inactive in buffer pH 10.0.

Results regarding the effect of various compounds on the r*Ps*Grx activity were shown in Table 1. Mg^2+^, Zn^2+^, Cu^2+^, Ni^2+^, and Ca^2+^ strongly inhibited the activity of r*Ps*Grx by 93.0, 94.2, 85.0, 83.0, and 82.8%, respectively. Addition of other metals, such as K^+^, Fe^2+^, Fe^3+^, and Mn^2+^, partially inhibited Grx activity. The presence of thiourea, DDT, Tween-80, SDS, and H_2_O_2_ decreased the activity, and EDTA kept Grx activity with 79.7%. It should be noted that 37.0% of the remaining activity was detected in the presence of high salt concentrations (1 M NaCl). Based on the Lineweaver-Burk plot, the *K*_*m*_ and *V*_max⁡_ values of rPsGrx using HED as substrate were 0.46 mM and 14.3 nmol/mL/min, respectively.

## 4. Discussion

Based on the phylogeny, sequence, and domain structure, Grxs have been discovered and identified in various species. In the present study, a novel Grx gene from* P*. sp. AN178 was cloned and expressed in* E. coli*. The 270bp* Ps*Grx gene with 89 amino acid (9.8 kDa) was similar to some of the other Grx genes such as* Chlorella virus* Grx (9.0 kDa) [18],* Trypanosoma cruzi* Grx (12.4 kDa) [19],* Taiwanofungus camphorata* Grx (11.0 kDa) [20], and* Panax ginseng* Grx (11.2 kDa) [21]. While larger molecular weight determination results to that of tomato* SlGrx1* (32.1 kDa) [22] and* E. coli* Grx2 (24.3 kDa) [23] have been reported, Grxs from different species may differ in their structures. Classical Grxs are 10 kDa proteins with a CPYC active site (Grx1 and Grx3 in* E. coli* and Grx1 and Grx2 in yeast). A second group with a CGFS active site corresponds to yeast Grx3, Grx4, and Grx5 [24]. The third type, represented by* E. coli* Grx2, is structurally related to the GST [25]. In this study,* Ps*Grx contains the N-terminal redox center C^12^ PFC^15^ sequence, which is typically CXXC motif present in Grx. Two cysteines in this motif were the source of reducing equivalents for substrate reduction. These residues formed reversible disulfide bond during catalysis and were shown to be the enzyme active site [1]. In addition,* Ps*Grx also contains conserved C-terminal sequences involved in GSH binding, which exhibited highest homology to other Grxs. Multiple sequence alignment of the full-length* Ps*Grx revealed that it shared the highest homologies with* P. haloplanktis* belonging to the TRX-like superfamily (Figure 1).

The optimum temperature for the r*Ps*Grx was observed at 30°C (Figure 3(a)). It was lower than the optimum temperature of* Chlorella virus* Grx (37°C) [17]. The previous studies have shown that Grxs are ubiquitous small heat-stable proteins.* Trypanosoma cruzi* Grx retained approximately 50% of activity at 100°C for 8.5 min [19];* Brassica campestris* Grx has no loss of activity at 95°C for 30 min [26], and* Cryptococcus neoformans* Grx was partially inactivated at 60°C or higher temperatures [27]. But r*Ps*Grx activity still showed 65.0% of its activity after incubation at 40°C for 20 min (Figure 3(b)), which displayed low thermal stability. It was known that the common features of the cold active enzyme are high catalytic activities at low temperatures and low thermal stability. These results indicated that r*Ps*Grx was a cold active protein. As shown in Figure 3(c), optimum activity was observed at pH 8.0. This result was consistent with Grx from* Brassica campestris* (pH 8.5) [26], Grx from* Oryza sativa* (pH 8.7) [28].

As shown in Table 1, 37.0% of its optimum activity was detectable at 1.0 M NaCl. This observation was found in other cold active enzymes from Antarctic sea-ice bacteria, such as 110.5, 96.7, and 81.4% of the GST, protease and lipase activity detected in the presence of 1.0 M concentrations, respectively [13, 29, 30]. It was sensitive to SDS and thiourea, indicating that hydrogen bonds played an important role in maintaining Grx activity. r*Ps*Grx was very sensitive to H_2_O_2_, and the similar result was described in the Grx from* Oryza sativa* [28], which could oxidize the reduced sulfhydryl groups. The *K*_*m*_ values of r*Ps*Grx (0.46 mM) were much lower than that of* Chlorella virus* Grx (1.4–2.1 mM) [4], human Grx2 (1.68 mM) [23], and* Cryptococcus neoformans* Grx (1.03 mM) [27], indicating r*Ps*Grx had a higher affinity for the substrate HED.

In conclusion, compared with other Grxs, r*P*sGrx displayed specific catalytic properties and was a typical cold active protein with low thermal stability. Further studies are undergoing to understand the physiological function of Grx in Antarctic sea-ice bacteria under environmental stresses.

## Figures and Tables

**Table 1:** Effect of various compounds on the r*Ps*Grx activity.

Reagent	Concentration	Relative activity (%)
None	—	100.0
DTT	10 mM	41.2
SDS	10 mM	11.3
Thiourea	10 mM	37.8
EDTA	10 mM	79.7
Tween-80	0.2%	53.1
Triton X-100	0.2%	19.0
H_2_O_2_	0.2%	9.2
Mg^2+^	5 mM	7.0
Zn^2+^	5 mM	5.8
Ca^2+^	5 mM	17.2
Cu^2+^	5 mM	15.0
Ni^2+^	5 mM	17.0
Mn^2+^	5 mM	39.5
Fe^2+^	5 mM	51.5
Fe^3+^	5 mM	51.3
K^+^	5 mM	26.8
Na^+^	0.5 M	69.1
Na^+^	1.0 M	37.0
Na^+^	1.5 M	11.2
